# Pain hypersensitivity mechanisms at a glance

**DOI:** 10.1242/dmm.011502

**Published:** 2013-07

**Authors:** Vijayan Gangadharan, Rohini Kuner

**Affiliations:** 1 Institute of Pharmacology, Heidelberg University, Im Neuenheimer Feld 366, 69120 Heidelberg, Germany; 2 Molecular Medicine Partnership Unit of the European Molecular Biology Laboratory and the University of Heidelberg Medical Faculty, Heidelberg, Germany

## Abstract

There are two basic categories of pain: physiological pain, which serves an important protective function, and pathological pain, which can have a major negative impact on quality of life in the context of human disease. Major progress has been made in understanding the molecular mechanisms that drive sensory transduction, amplification and conduction in peripheral pain-sensing neurons, communication of sensory inputs to spinal second-order neurons, and the eventual modulation of sensory signals by spinal and descending circuits. This poster article endeavors to provide an overview of how molecular and cellular mechanisms underlying nociception in a physiological context undergo plasticity in pathophysiological states, leading to pain hypersensitivity and chronic pain.

## Introduction

Pain is an important, evolutionarily conserved physiological phenomenon that is necessary for survival. At the same time, pain is one of the most frequent symptoms of a variety of pathological disorders and represents a major clinical challenge. In recent decades, there has been a dramatic increase in our understanding of molecular and cellular mechanisms underlying pain in physiological, as well as pathophysiological, contexts. A clearer picture is beginning to emerge out of the myriad signaling pathways that have been implicated in disease-related pain hypersensitivity.

Noxious stimuli, including mechanical, chemical and thermal stimuli, are sensed by peripheral nociceptive neurons that are classified as C or A-delta (Aδ) type based on properties of the nerve fiber. A third type, A-beta (Aβ) fibers, are involved in the conduction of non-nociceptive inputs such as light touch, movement or vibration under normal physiological conditions. Morphological, electrophysiological and genetic studies have provided evidence for specificity of peripheral nociceptive and non-nociceptive sensory neurons for distinct sensory modalities. The somata or cell bodies of both nociceptive and non-nociceptive sensory afferents lie in the dorsal root ganglia (DRG), and their central terminals synapse in the superficial spinal dorsal horn. Spinal circuits further process sensory inputs and relay them to brain centers via diverse pathways, where the perception of pain together with its emotional and aversive components is generated. In this review, we will outline key mechanistic events and attempt to derive common principles and potential therapeutic windows from the abundant literature that is available.

## Peripheral signaling pathways involved in acute and chronic pain

### Receptors involved in nociception

The diverse range of ion channels that is present on sensory nerve endings mediates the transduction of physicochemical stimuli into changes in membrane potential (see Poster, panel A). Warm and hot temperatures are sensed by transient receptor potential (TRP) channels such as TRPV1 and TRPV2, and also by a calcium-gated chloride (Ca^2+^-gated Cl^−^) channel, ANO1 (Cho et al., 2012; Julius and Basbaum, 2001). Protons are detected by acid-sensing channels (ASICs) and also by TRPV1 (Julius and Basbaum, 2001). TRPM8 is the sensor for cold temperatures, and Nav1.8 (described below) is required for cold-associated pain (Bautista et al., 2007; Zimmermann et al., 2007). Piezo1 and Piezo2 are thought to act as mechanical transducers (Coste et al., 2010), although TRPA1 and the ATP-gated purinergic ion-channel P2X3 have also emerged as mediators of mechanical hyperalgesia (Kwan et al., 2006; Petrus et al., 2007; Tsuda et al., 2000). Activation of these ion channels leads to the generation of a transient potential, which is amplified in the form of a ‘regenerative potential’ by sodium (Na^+^) channels such as Nav1.8 and Nav1.9 (Raouf et al., 2010). At this stage, the signal can be modulated by endogenous inhibition, which occurs via recruitment of potassium (K^+^) channels such as the two-pore channels TREK1 and TRAAK1 (Honoré, 2007). Finally, the activation of other Na^+^ channels, such as Nav1.7, triggers an action potential that carries nociceptive information from the peripheral nervous system into the central nervous system (CNS) (Raouf et al., 2010; Wood et al., 2004). A loss-of-function mutation in the human *Nav1.7* gene reportedly leads to complete insensitivity to pain (Cox et al., 2006). Conversely, a gain-of-function *Nav1.7* mutation causes congenital paroxysmal extreme pain disorders; for example, erythromelalgia (Fertleman et al., 2006). In line with these clinical observations, nociceptor-specific deletion of *Nav1.7* leads to decreased pain hypersensitivity in mice (Nassar et al., 2004). Moreover, a Na^+^-channel blocker has been shown to be effective in relieving spontaneous pain in individuals with erythromelalgia (Goldberg et al., 2012).

### Peripheral sensitization

In states of chronic pain, particularly in the context of inflammation and cancer, nociceptive and non-nociceptive sensory afferents are sensitized. Peripheral sensitization represents a reduction in the threshold and/or an increase in magnitude of responsiveness at the peripheral ends of sensory nerve fibers. This occurs in response to chemical mediators released by nociceptors and non-neuronal cells (e.g. mast cells, basophils, platelets, macrophages, neutrophils, endothelial cells, keratinocytes and fibroblasts) at the site of tissue injury or inflammation. A wide range of signaling molecules is involved in mediating peripheral sensitization, including protons, ATP, prostaglandins (PGE2), thromboxanes, leukotrienes, endocannabinoids, growth factors such as neurotrophins [nerve growth factor (NGF)] and granulocyte- or granulocyte-macrophage colony stimulating factors (G-CSF, GM-CSF), cytokines (IL6, IL1β, TNFα), chemokines, neuropeptides [calcitonin gene-related peptide (CGRP), substance P, bradykinin, histamine], lipids, and diverse proteases (Binshtok et al., 2008; Gold and Gebhart, 2010; Julius and Basbaum, 2001; Schweizerhof et al., 2009). Interestingly, glutamate, which is the major excitatory synaptic transmitter at central synapses, contributes to sensitization at peripheral nerve endings by binding in a non-synaptic manner to AMPA and NMDA receptors (AMPAR and NMDAR, respectively) to mediate peripheral cell-cell interactions (e.g. upon release from immune cells) or autocrine regulation (e.g. upon release from sensory endings following TRPV1-mediated Ca^2+^ influx) (Gangadharan et al., 2011).

**Figure:**
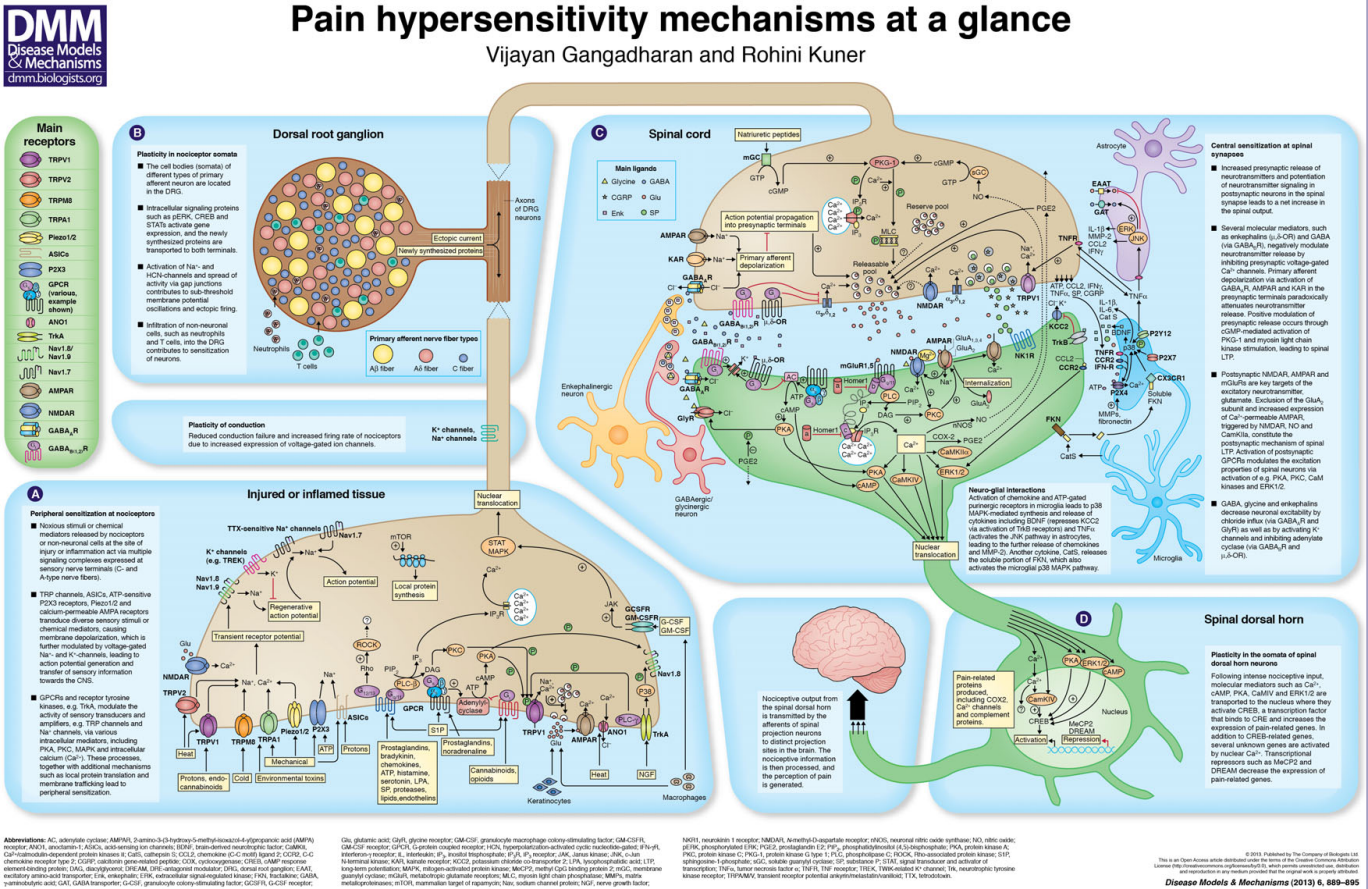


In response to signals from chemical mediators, the activity of transduction proteins that control the excitability of nociceptor terminals is modulated at the transcriptional or post-translational level. For example, growth factors (such as NGF acting via the tyrosine kinase receptor TrkA, or GM-CSF acting via tyrosine kinase receptors and JAK-STAT signaling), recruit multiple downstream enzymes, including phospholipase C (PLC), phosphoinositide 3-kinase (PI3K) and mitogen-activated protein kinase (MAPK). These enzymes not only directly phosphorylate transducer and ‘amplifier’ molecules, such as TRPV1 and Nav1.8, but also enhance the expression of these molecules via transcriptional regulation (e.g. via recruitment of STAT transcription factors), thus leading to an acute as well as a long-term increase in the excitability of nociceptors (Dib-Hajj et al., 1998; Julius and Basbaum, 2001; Schweizerhof et al., 2009; Zhang et al., 2005).

### The role of G-protein coupled receptors

G-protein coupled receptor (GPCR) signaling also plays a role in peripheral sensitization. A diverse set of peptides, metabolic products and bioactive lipids activate GPCRs in sensory neurons. These GPCRs are capable of coupling to different G-proteins: G_q_, G_11_, G_s_, G_i_, G_12_ or G_13_. The G_q_/G_11_ signaling branch mediates the activation of phospholipase C-β (PLC-β) and protein kinase C (PKC), the release of Ca^2+^ from intracellular stores, and modulation of extracellular regulated kinases (ERK1, ERK2) (Julius and McCleskey, 2005; Kuner, 2010). The G_s_ signaling branch, by contrast, is linked to cAMP–protein-kinase-A (PKA)-mediated sensitization mechanisms (Hucho and Levine, 2007). Recent results indicate that the functional role of the G_q_/G_11_ signaling branch in nociceptors *in vivo* not only spans sensitization mechanisms in pathological pain states but also covers basal nociception and acute pain (Tappe-Theodor et al., 2012). This includes tonic modulation of TREK channels (Chen et al., 2006), Na^+^ channels (Tappe-Theodor et al., 2012), TRPV1 (Zhang et al., 2012) and mechanosensory currents (Lechner and Lewin, 2009). Most GPCRs that signal via G_q_ and G_11_ also couple to G_12_ and G_13_ proteins, which are capable of activating the RhoGTPase RhoA and, in turn, a downstream kinase, ROCK. However, the significance of RhoA-ROCK signaling in nociception is not known. Finally, G_i_-mediated inhibition constitutes an important checkpoint in determining nociceptor excitability. The anti-nociceptive actions of cannabinoids and opioids, which bind to GPCRs, are also mediated via peripheral mechanisms (Agarwal et al., 2007; Kinsey et al., 2009; Lever et al., 2009; Stein and Lang, 2009).

## Signaling events in nociceptor somata

In response to persistent nociceptive activity in peripheral tissues, several synapse-to-nucleus messengers are recruited. These include STAT3, MAPKs, e.g. ERK1 or ERK2, and cAMP-PKA, which drive activity-dependent transcription (Woolf and Costigan, 1999). However, the functional role of the somata of sensory neurons goes far beyond sustaining neuronal survival and synthesizing proteins that impart or modulate cell function. The somata of DRG neurons have evolved as the seat of intriguing mechanisms governing aberrant excitation and cell-cell interactions in chronic pain models (see Poster, panel B). For example, they are involved in the generation of ectopic discharges and oscillatory activity in states of neuropathic pain (Devor, 2006), e.g. by recruiting hyperpolarization-activated cyclic nucleotide-gated ion-channels (HCN) channels (Emery et al., 2011). Furthermore, gap junctions on satellite cells surrounding the somata of sensory neurons have also been implicated in the spread of aberrant excitation within the DRG (Zhang et al., 2009). Another intriguing recent finding is that non-neuronal cells, such as neutrophils and T cells, invade the DRG in inflammatory and neuropathic pain states (Kim and Moalem-Taylor, 2011). However, the identity and significance of these cell-cell interactions is not yet clear.

## Central signaling pathways involved in acute and chronic pain

### Postsynaptic mechanisms

Excitatory synaptic communication between primary afferents and spinal neurons is mediated primarily by glutamate, with modulatory influences from co-transmitters such as substance P, CGRP and brain-derived growth factor (BDNF). Both ionotropic and metabotropic (mostly G_q_- or G_11_-coupled) glutamatergic receptors play a key role in determining the strength of synaptic transmission and modulations in the spinal cord following persistent nociceptive activity (see Poster, panel C). Activity-dependent changes in spinal function encompass long-term potentiation (LTP) of individual synapses as well as an increase in neuronal and non-neuronal excitation in the spinal dorsal horn, leading to increased pain sensitivity, i.e. central sensitization (Ji et al., 2003; Sandkühler, 2009). A key trigger for both types of change is the activation of spinal postsynaptic NMDARs following persistent nociceptive activity (Woolf and Salter, 2000). The ensuing rise in intracellular Ca^2+^ activates protein kinases, such as CaMKIIα, that bring about the insertion of greater numbers of AMPA-type glutamate receptors in postsynaptic membranes via recruitment of a variety of AMPAR-interacting proteins, such as GRIP1 (Kuner, 2010). This not only enhances postsynaptic excitation, but also leads to further influx of Ca^2+^ via the recruitment of Ca^2+^-permeable AMPAR (Galan et al., 2004; Hartmann et al., 2004; Park et al., 2009). Additional Ca^2+^- dependent kinases are also activated, such as cyclooxygenases (COX-2) and nitric oxide synthases (NOS), which generate prostaglandin E2 and nitric oxide, respectively. These molecules have been proposed to function as retrograde messengers, facilitating neurotransmitter release from primary afferent terminals in the spinal dorsal horn. A variety of synaptic-interacting proteins come into play to optimally position NMDAR and AMPAR channels in the postsynaptic membrane (Kuner, 2010). Interestingly, persistent nociceptive activity also recruits synaptic proteins that counteract or inhibit central sensitization, either by inhibiting key enzymes, e.g. NOS-interacting protein, or by disassembling complexes of metabotropic glutamate receptors 1 and 5 (mGluR1,5) together with inositol triphosphate receptors (IP_3_R) that guard intracellular Ca^2+^ stores. The MAPKs ERK1 and ERK2 are also activated downstream of glutamatergic ion channels and GPCRs. These MAPKs directly regulate the excitability of spinal neurons by modulating the K_v_4.2 channel, which generates A-type K^+^ currents that regulate neuronal excitability. Phosphorylation of the K_v_4.2 channel by ERK1/2 decreases A-type currents and increases excitability of superficial spinal cord dorsal horn neurons (Hu et al., 2006). In addition, ERK1 and ERK2 have been shown to enhance AMPAR- and NMDAR-mediated currents in spinal cord neurons (Kohno et al., 2008).

### Presynaptic mechanisms

Although most of the research on spinal mechanisms of chronic pain has focused on postsynaptic mechanisms in spinal neurons, recent studies also indicate prominent presynaptic plasticity. For example, LTP at synapses between nociceptors and spinal neurons projecting to the periaqueductal gray requires postsynaptic NMDAR activation for induction (Ikeda et al., 2006), but also recruits a cGMP-driven increase in presynaptic neurotransmitter release (Luo et al., 2012). This is brought about by presynaptic activation of protein kinase G1 (PKG1), which phosphorylates presynaptic IP_3_Rs as well as myosin light chain (MLC) subunits, resulting in a Ca^2+^-evoked increase in actin-myosin coupling and recruitment of synaptic vesicles from reserve pools (see Poster, panel C). Interestingly, nerve injury itself is associated with an increase in neurotransmitter release from nociceptors (Inquimbert et al., 2012).

### Genes underlying chronic pain

A major question in the investigation of pain pertains to how the transition between acute sensitization and long-lasting, persistent pain comes about. Activation of genomic programs that harness a chronic ‘memory’ component of pathological pain is considered to be a key mechanism. In this context, ERK1, ERK2, cAMP and CaMKIV function as synapse-to-nucleus communicators to trigger the activation of the cAMP response element-binding protein (CREB), which drives the expression of a variety of pain-related proteins, such as COX-2, TRPV1 and Ca^2+^ channels, among others (see Poster, panel D) (Kawasaki et al., 2004). Intriguingly, Ca^2+^ was recently found to travel into the nucleus of spinal excitatory neurons in a nociceptive activity-dependent manner, driving a unique genomic program that regulates both functional and structural plasticity in inflammatory pain (Simonetti et al., 2013). Gene transcription in spinal neurons is also regulated by activity-dependent expression of transcriptional repressors such as MeCP2, which regulates mTOR signaling (Géranton et al., 2007), and DREAM, which acts constitutively to suppress prodynorphin expression in spinal cord neurons and to thereby elicit hyperalgesia (Cheng et al., 2002).

### Inhibition and disinhibition of central sensitization

Persistent nociceptive activity-induced pronociceptive drive and central sensitization are held in check by spinal inhibitory networks, comprising GABAergic and glycinergic neurotransmission (see Poster, panel C, lower left). Endogenously released cannabinoids, opioids and adenosine also play an inhibitory role. For example, local enkephalins (released by enkephalinergic neurons) inhibit neurotransmitter release and depress postsynaptic excitation, via G_i_-mediated inhibition of voltage-gated Ca^2+^ channels and G_i_-mediated activation of GIRK-type K^+^ channels, respectively. These enkephalinergic inhibitory mechanisms are recruited spinally by descending serotonergic and noradrenergic systems, constituting brainstem control of central sensitization. There are also several molecular signaling events that are associated with disinhibition after nerve injury. For instance, PGE2 inhibits PKA-mediated phosphorylation of the α3 subunit of the glycine receptor, thereby counteracting glycinergic inhibition (Harvey et al., 2004). Another mechanism for disinhibition of spinal neurons is nerve-injury-induced collapse of the Cl^−^ gradient, brought about by a loss of the postsynaptic potassium chloride (K^+^ Cl^−^) exporter KCC2, which ultimately results in reduced generation of GABA-mediated inhibitory postsynaptic currents (Beggs et al., 2012; Coull et al., 2003).

## Signaling events associated with neuro-glia interactions

In recent years, signaling mechanisms that mediate interactions between spinal neurons and diverse types of glial cells have been uncovered at an amazing pace. Purinergic signaling, involving P2X4 (Beggs et al., 2012), P2X7 (Clark et al., 2010a; Clark et al., 2010b) and P2Y12 (Tozaki-Saitoh et al., 2008) receptors, plays a central role in the recruitment and activation of microglia, which have emerged as key regulators of central sensitization (see Poster, panel C, right-hand side) (Gao and Ji, 2010a; McMahon and Malcangio, 2009). A great deal of interest has been focused on understanding the intracellular signaling pathways in activated microglia. Following nerve injury, chemokines released from the primary afferent terminals, such as CX3CL1, CCL2 and TNFα, as well as ATP, activate their cognate receptors on microglia (Beggs et al., 2012; Clark et al., 2010a; Gao and Ji, 2010a). Activation of these receptors induces the p38 MAPK signaling pathway in microglia, which is believed to underlie the synthesis and release of a variety of molecular mediators, such as BDNF, TNFα, IL-1β, IL-6 and cathepsin S, that alter neuronal function (Clark et al., 2009; Kawasaki et al., 2008). BDNF released from microglia acts on TrkB receptors in postsynaptic neurons to downregulate the expression of the potassium chloride co-transporter KCC2 in neighboring neurons, thereby rendering them more prone to excitation (Coull et al., 2003). Microglia-derived TNFα activates the JNK pathway in astrocytes, leading to the further release of IL-1β, CCL2 and MMP-2, which modulate central sensitization. TNFα was also shown to activate TNFR on presynaptic terminals, leading to the release of glutamate and increased excitatory postsynaptic potential (EPSP) via TRPV1 activation (Park et al., 2011). CCL2 and IL-1β released from astrocytes bind to their receptors (CCR2 and ILR, respectively) at pre- and postsynaptic sites, leading to increased neurotransmitter release and enhanced activation of NMDAR and AMPAR (Gao and Ji, 2010b). MMP-9 and MMP-2 induce cleavage and activation of IL-1β, leading to further activation of microglia and astrocytes, thereby contributing to the development and maintenance of neuropathic pain. Cathepsin S released from microglia cleaves a transmembrane protein, fractalkine (FKN), that is expressed in spinal dorsal horn neurons and leads to the release of soluble FKN (s-FKN) that binds to its receptor, CX3CR1, on the microglia. This again triggers the activation of the p38 MAPK signaling pathway in microglia, establishing positive feed-forward and feedback modulatory loops that probably contribute to the maintenance of chronic pain long after the initial injury is triggered. In chronic pain conditions, astrocyte activation also leads to negative and positive modulation of EAAT and GABA transporters, respectively. This results in increased availability of excitatory amino acids such as glutamate and decreased availability of the inhibitory neurotransmitter GABA, and therefore in increased synaptic transmission.

## Potential therapies to combat pain

Despite substantial advances in pain research, the barriers in developing novel therapeutics remain enormous. There are several difficulties associated with taking forward a drug target from bench to bedside. First, key mediators of nociceptive processing are not specific and have global functions in normal physiology (e.g. PLC, CREB and MAPK); second, the existence of redundant mechanisms and mediators in pain pathways makes it difficult to select suitable drug targets. To date, the majority of drugs that have been used to treat patients target mechanisms that have been known for several years. For example, traditional anti-inflammatory drugs such as salicylic acid, paracetamol, opioids and non-steroidal anti-inflammatory drugs (NSAIDs) remain the major players for the treatment of pain. Although these drugs are generally effective, the most efficacious ones among them are associated with unpleasant side effects such as nausea, vomiting, and renal and cardiovascular complications. Unfortunately, the newly developed COX-2 inhibitors also failed to be adopted clinically owing to exacerbation of cardiovascular side effects (Mukherjee et al., 2001; McGettigan and Henry, 2006).

Recently, clinical trials were launched for Tanezumab and other monoclonal antibodies that act against NGF. Results from these trials suggest that anti-NGF therapy could represent an important new class of therapy for pain management in chronic pain conditions. Although promising, this novel approach is not, however, devoid of side effects (McKelvey et al., 2013).

There are also several ion channel inhibitors that provide a novel therapeutic approach for the treatment of pain. Procaine, bupvacaine and lidocaine are voltage-gated Na^+^ channel blockers that are effective anti-nociceptives upon local application, but their efficacy is limited to disorders with ongoing peripheral nociceptive activation (e.g. postherpetic neuralgia), rather than central pain disorders (conditions caused by damage to or dysfunction of the CNS). Other Na^+^ channel blockers, such as carbamezapine and lamotrigin, are efficacious against trigeminal neuralgia pain. The development of subtype-specific Na^+^ channel inhibitors is another strategy being implemented by drug companies, which holds tremendous promise. Ziconotide, a synthetic peptide that blocks presynaptic N-type voltage-gated Ca^2+^ channels and interferes with neurotransmitter release, is highly efficacious in individuals with chronic pain; however, its use is limited by CNS side effects. In addition, the drug must be given intrathecally to circumvent cardiac dysfunction. Leconotide is an alternative to conopeptides such as Ziconotide, and has been suggested to have fewer CNS side effects (Kolosov et al., 2010). Pregabalin and gabapentin, which are moderately effective in relieving neuropathic pain, have been proposed to target accessory α2δ subunits of Ca^2+^ channels, although the supporting data are highly controversial. Recent studies indicate that gabapentin blocks spine morphogenesis, thereby implicating an alternative mechanism (Eroglu et al., 2009). Newly developed TRPV1 antagonists are also promising, yet they have been found to profoundly affect core body temperature, impeding their use in treating chronic pain disorders (Gavva et al., 2008).

Finally, there has been a substantial amount of interest in developing drugs that interfere with the interaction between nonneuronal populations and pain-processing neurons, based upon recent evidence of the roles played by non-neuronal cells (e.g. microglia and astrocytes) in the development and maintenance of chronic pain. Ongoing clinical trials will shed light on the potential druggability of these interactions, in addition to other newly discovered targets. Given the complexity and diversity of pain conditions, it is clinically very important to develop site-specific delivery tools as well as mechanism-based drugs.

## Summary and outlook

As outlined in this review, tremendous progress has been made in understanding the neurobiology of peripheral and central sensitization in sensory-afferent–spinal-cord circuits that process nociception. This rich diversity of mediators provides enormous scope for drug discovery in the context of pain therapeutics. By contrast, much remains to be understood about mechanisms driving plasticity and reorganization in cortical circuits, where the perception of pain is generated. This remains a major challenge to tackle in the coming years.
